# Comprehensive Analysis of DGATs and PLINs in Ovarian Cancer: Implications for Diagnosis and Prognosis

**DOI:** 10.1155/bmri/9153643

**Published:** 2025-10-01

**Authors:** Vijayalakshmi N. Ayyagari, Miao Li, Paula Diaz-Sylvester, Kathleen Groesch, Teresa Wilson, Zvi Pasman, Laurent Brard

**Affiliations:** ^1^ Division of Gynecologic Oncology, Department of Obstetrics and Gynecology, Southern Illinois University School of Medicine, Springfield, Illinois, USA, siu.edu; ^2^ Simmons Cancer Institute, Southern Illinois University School of Medicine, Springfield, Illinois, USA, siu.edu; ^3^ Center for Clinical Research, Southern Illinois University School of Medicine, Springfield, Illinois, USA, siu.edu; ^4^ Department of Chemistry, Illinois College, Jacksonville, Illinois, USA

**Keywords:** DGATs, diagnostic models, lipid droplets, ovarian cancer, PLINs, prognostic

## Abstract

**Background:**

Lipid droplet (LD) dynamics drive cancer cell proliferation, resistance, and aggressiveness. Diacylglycerol O‐acyltransferases (DGATs) and perilipins (PLINs) are key LD‐associated genes implicated in cancer pathophysiology.

**Objective:**

This study aimed to comprehensively analyze the expression and clinical significance of DGATs and PLINs in ovarian cancer (OC), focusing on their correlation with LDs and triglyceride (TG) levels, and to explore their diagnostic and prognostic implications.

**Methods:**

LD and TG levels in ovarian cell lines and clinical samples were assessed using BODIPY staining, fluorometric, colorimetric assays, and thin‐layer chromatography (TLC). Gene expression profiling of DGATs and PLINs in cell lines and tissue was conducted via RT‐qPCR, ELISA, and bioinformatics analysis. Correlation analyses between gene expression, Ki67, and survival data were performed. ROC curve analysis evaluated diagnostic potential.

**Results:**

LD accumulation was significantly higher in OC cell lines and tissues compared with normal controls. Diacylglycerol O‐acyltransferase 1 (DGAT1) and diacylglycerol O‐acyltransferase 2 (DGAT2) were overexpressed in OC cell lines and tissues, particularly in advanced stages (III and IV). Elevated TG levels were observed in OC cell lines and clinical samples, correlating with LD abundance and the expression of DGAT1 and DGAT2. PLIN2 and PLIN3 were significantly upregulated in OC tissues. Bioinformatics analysis identified dysregulation of DGATs and PLINs in OC. Survival analysis indicated DGAT2 is a predictor of poor prognosis. Diagnostic assessments revealed DGAT2 as a potential biomarker for OC detection.

**Conclusion:**

DGATs and PLINs are pivotal in LD metabolism and tumor progression in OC, with DGAT2 being a good candidate as prognostic and diagnostic marker. They present promising avenues for therapeutic targeting and diagnostic biomarkers, holding the potential to improve patient outcomes. Further exploration of their mechanistic roles and clinical implications is essential for advancing personalized cancer care.

## 1. Introduction

Ovarian cancer (OC) is often diagnosed in advanced stages, representing a significant clinical challenge. Despite initial success of treatments, such as cytoreductive surgery and platinum‐based chemotherapy, approximately 70% of patients with advanced OC experience relapse within 1 to 2 years posttreatment [1, 2]. This emphasizes the urgent need for advancements in understanding the molecular mechanisms underlying this disease, crucial for improving prognosis and early diagnosis.

The tendency of OC to metastasize to omental adipocytes indicates the involvement of the fatty acid (FA)–enriched tumor microenvironment in tumor progression [3]. Metabolic alterations favoring lipid accumulation stimulate the formation of lipid droplets (LDs), which play crucial roles in lipid balance, energy metabolism, and cellular signaling [4, 5]. The abundance of LDs correlates with tumor aggressiveness, chemotherapy resistance, and poor clinical outcomes across various cancers, underscoring LDs as a potential therapeutic target for OC [6, 7].

While LDs function as organelles, their biosynthesis, degradation, and other functions depend primarily on associated proteins [8–11]. Numerous studies have explored these proteins, revealing their differential expression in specific cancer types or their correlation with tumor stages and outcomes, suggesting their diagnostic, prognostic, and therapeutic potential in clinical practice [12, 13]. Moreover, functional in vitro and in vivo experiments have elucidated their impact on cancer phenotypes such as proliferation, metastasis, and chemotherapy resistance, suggesting their utility as therapeutic targets [5, 6, 13, 14]. LD proteomics studies have identified multiple key proteins involved in lipid metabolism. This study focuses on proteins most directly impacting LD biosynthesis and function.

LDs are formed de novo following the synthesis of triglycerides (TGs) and cholesterol esters (CEs) by endoplasmic reticulum (ER) resident diacylglycerol O‐acyltransferases (DGATs) and acyl‐CoA cholesterol acyltransferases (ACATs), respectively [4–7]. TG and CE constitute the key neutral lipid core of LDs. Similarly, proteins of the perilipin (PLIN) family associated with LD surfaces also contribute to LD formation and function [5, 11–13]. In numerous cancers, the accumulation of LDs is frequently associated with altered expression of DGATs, ACAT1, and PLINs, indicating their pivotal role in cancer progression [15–21]. Although previous research has offered valuable insights into the significance of these proteins in cancer biology, a comprehensive analysis specific to OC is currently lacking. This study aimed to evaluate these key LD‐associated genes in both ovarian cell lines and clinical samples to understand their association with malignancy, tumor aggressiveness, and patient survival. We conducted a thorough analysis of DGATs and PLINs, including examining their expression levels, identifying major isoforms, and investigating their association with LD accumulation in ovarian cell lines. Clinical validation was performed by assessing these factors in diverse biological samples, such as OC tumor tissue, peritoneal fluid (PF), and plasma, to determine their prognostic, diagnostic, and/or therapeutic potential. Employing a multidisciplinary approach that integrates experimental techniques with bioinformatics analyses, this study sought to provide an extensive understanding of the role of LD‐associated proteins, particularly DGATs and PLINs, in OC.

## 2. Materials and Methods

### 2.1. Cell Lines and Chemicals

Human primary ovarian epithelial cells (H‐6036) were purchased from Cell Biologics (Chicago, IL, United States) and human fallopian tube epithelial cells (HFTECs) from Lifeline Cell Technology (United States). The ovarian carcinoma cell line NIH‐OVCAR3 was obtained from ATCC, and the SKOV‐3 human ovarian carcinoma cell line was provided by Dr. Mary McAsey′s laboratory at SIU School of Medicine in Springfield, IL. Isogenic OC cell line pairs, namely, A2780/A2780‐CDDP and IGROV‐1/IGROV‐1CDDP, were acquired from Dr. Alexander Brodsky at Brown University, Providence, RI. Growth media and conditions were as previously described [20, 22], and all cell lines were maintained under identical culture conditions to ensure consistency across experiments. All cell lines were authenticated prior to experimental use and confirmed to be free of mycoplasma contamination.

DGAT1 and DGAT2 inhibitors, A922500 and PF‐06424439, respectively, were purchased from Millipore Sigma, United States. Antibodies for DGAT1, DGAT2, and PLINs were obtained from Novus Biological (United States). Ki67 antibody was purchased from Abcam (Cambridge, MA, United States). DGAT1 RTU enzyme‐linked immunosorbent assay (ELISA) kit (Catalog # MBS160541) and DGAT2 ELISA kit (Catalog # MBS458842) were purchased from MyBioSource.

### 2.2. Ethic Statement, Standard Protocol Approvals, and Patient Consent

As described earlier [21], this observational, cross‐sectional pilot study was conducted at Southern Illinois University′s Department of Obstetrics and Gynecology, Division of Gynecological Oncology from 2016 to 2021. This study was approved by the SIU Medicine Institutional Review Board under protocols 16‐493 and 12‐656. Excluding those with previous malignancies or oncologic treatments, eligible patients, aged 30 and above, were approached during their preoperative evaluation. All participants provided written informed consent prior to enrollment.

### 2.3. Clinical Samples

As described earlier [21], patients scheduled for gynecologic surgery for suspicion of OC presenting to the Division of Gynecological Oncology and those with benign conditions (e.g., uterine prolapse) from the General Gynecology and Urogynecology divisions were consented and enrolled. Samples were collected on the day of surgery. Post‐surgery, patients were divided into three groups based on final pathology: OC (*N* = 32), benign pelvic masses (BPMs) (*N* = 18) and normal ovaries (*N* = 14). The BPM and normal groups were combined into a nonmalignant cohort, referred as the control group (*N* = 32) for comparison with the OC group. Patient data including age, menopausal status, cancer stage, and comorbidities were obtained from the electronic medical records and summarized in Table 1.

**Table 1:** Clinical and pathological characteristics of samples.

**Parameter**	**Nonmalignant**	**OC**
Sample size, *n* (%)	32 (50)	32 (50)
Age, median years (min–max)	64 (38–82)	60 (48–82)
BMI, median kg/m^2^ (min–max)	27.82 (19–52)	29 (18–47)
Premenopausal, *n* (%)	5 (19)	2 (6)
Postmenopausal, *n* (%)	23 (85)	30 (94)
Obesity, *n* (%)	7 (26)	8 (26)
Diabetes, *n* (%)	7 (26)	6 (19)
Hypertension, *n* (%)	11 (41)	17 (23)
Hypothyroidism, *n* (%)	8 (30)	4 (13)
FIGO stage, *n* (% of OC)		
Stage I		8 (25)
Stage II		4 (13)
Stage III		18 (56)
Stage IV		2 (6)
FIGO grade, *n* (% of OC)		
Low grade		6 (19)
High grade		22 (69)
No information		4 (13)
Histotype, *n* (% of OC)		
Endometrioid		2 (6)
Serous		24 (75)
Mucinous		4 (13)
Others		2 (6)

### 2.4. Peripheral Blood, PF, and Tumor Tissue Sample Collection

Peripheral blood was collected into sodium heparin tubes before surgery, and PF was obtained during surgery as previously described [21], involving aspiration of ascites, saline infusion, and re‐aspiration. Both plasma and PF samples were centrifuged at 1500 r/min for 10 min and stored at −80°C. Tissue fragments (≥ 1 cm^3^) free of necrosis were collected from the ovaries post‐oophorectomy, with macro‐dissection performed to isolate tumor or normal tissue, which was then flash‐frozen in liquid nitrogen and stored at −80°C until analysis.

### 2.5. RNA Extraction and cDNA Synthesis

RNA was extracted from cell lines, tumor, and normal ovarian tissues as described previously [21, 23] using TRIzol Reagent (Invitrogen; Thermo Fisher Scientific, Inc.). Subsequently, the yield and quality of the RNA were assessed before storing it at −80°C. From each sample, 1 *μ*g of RNA was reverse transcribed into cDNA using the iScript cDNA synthesis kit (Bio‐Rad).

### 2.6. Gene Expression Analyses by Reverse Transcription‐Quantitative Polymerase Chain Reaction (RT‐qPCR)

mRNA expression levels of key LD‐associated genes were quantified using RT‐qPCR with specific primers obtained from Integrated DNA Technologies, Inc. The procedure was performed as described previously [21, 23]. RPl4 and *β*‐actin served as housekeeping genes for normalization. The analysis utilized PowerUp SYBR‐Green Master Mix (Applied Biosystems; Thermo Fisher Scientific, Inc.) and was performed on the Applied Biosystems 7500 Real‐Time PCR System (Applied Biosystems; Thermo Fisher Scientific, Inc.). Measurements were conducted in triplicate, and relative mRNA levels were calculated using the *ΔΔ*Ct method. The primers for DGAT1, DGAT2, PLINs 1‐5, RPL4, and *β*‐actin are provided in Table S1.

### 2.7. Western Blot Analysis

Western blotting was performed to evaluate the expression of DGAT1 and DGAT2 proteins in cell lines. Cell seeding, cell lysis, and western blotting were done as described previously [20, 22]. Primary antibodies were diluted as follows: Anti‐DGAT (1:500), Anti‐DGAT2 (1:500), and anti‐*β*‐actin (1:1000).

### 2.8. Quantification of DGATs by ELISA

DGAT1 and DGAT2 protein levels in cell lysates, plasma, PF, and tissue lysates were quantified using ELISA kits from MyBioSource (San Diego, CA, United States). In cell lines, 4 × 10^5^ cells were plated in a six‐well plate and incubated for 48 h at 37°C in a 5% CO_2_ humidified atmosphere. Post‐incubation, cell lysate was prepared using lysis buffer consisting of 1% Triton X‐100, 150 mM NaCl, 50 mM Tris‐HCl, 1 mM EGTA, and 0.1% sodium dodecyl sulfate. Regarding clinical samples, tissue lysates for ELISA were prepared as described previously [21, 23]. In brief, tissues were homogenized in the lysis buffer mentioned above supplemented with 1 mM PMSF and 1X complete protease inhibitor (A32955, Thermo Fisher Scientific, MO, United States), and then sonicated. ELISA was performed following the manufacturer′s instructions. Absorbance was recorded at 450 nm using a Synergy H1 microplate reader (BioTek, VT, United States). Results were presented as *m* *e* *a* *n* ± standard deviation (SD) from three replicates. Comparative analyses were conducted between OC and controls. A bicinchoninic acid (BCA) protein assay kit (Bio‐Rad, United States) was utilized to assess protein concentration. DGAT concentration in plasma and PF were determined in 50 *μ*L aliquots of the sample following the kit manufacturer′s instructions.

### 2.9. BODIPY 493/503 Staining

Cells were treated with 1 *μ*M BODIPY 493/503 (Molecular Probes) for 15 min at room temperature, washed with PBS, and fixed in 4% paraformaldehyde at 4°C for 10 min. After PBS wash, cells were counter stained with NucBlue (Molecular Probes) and visualized using an Olympus DP73 fluorescence microscope. Flow cytometry analysis was performed using an Accuri C6 cytometer equipped with the FCSexpress software (BD Biosciences).

### 2.10. Quantitative Analysis of TG From Cell Lines and Tissue Samples

TG quantification was performed using a Colorimetric Assay Kit (Elabscience, United States), following the manufacturer′s guidelines. In cell lines, 1 × 10^6^ cells were homogenized in 300–500 *μ*L of PBS (0.01 M, pH 7.4) using an ultrasonic cell disruptor at 4°C. The homogenate was then centrifuged at 12,000 × g for 10 min at 4°C to precipitate insoluble material. TG levels were estimated from the supernatant. For tumor tissue, 20 mg tissue was homogenized in 180 *μ*L buffer at 4°C, centrifuged, and the supernatant was collected on ice for analysis. Protein concentration in the supernatant was determined, and TG levels were estimated according to kit instructions.

### 2.11. Bioinformatics Analysis

The mRNA expression levels of DGATs and PLIN family genes in OC and normal tissues were analyzed using the GEPIA2 platform (http://gepia2.cancer-pku.cn/#index). Mutations and copy number alterations in DGAT and PLIN family genes in OC were examined using The Cancer Genome Atlas Program (TCGA) datasets accessed through the cBioPortal databases (https://www.cbioportal.org/) for Cancer Genomics.

In OC, the association between mRNA expression levels of each individual gene and overall survival (OS) of patients was investigated using the Kaplan–Meier plotter (http://www.kmplot.com). Patients were categorized into “low” and “high” expression groups based on the median mRNA levels of each gene. Survival analysis was performed using the Kaplan–Meier method and the log‐rank test for univariate OS analysis. Hazard ratios (HRs), 95% confidence intervals (CIs), and log‐rank *p* values were calculated, with a *p* value of < 0.05 deemed statistically significant.

### 2.12. Statistical Analysis

The clinical/pathological variables and comorbidities were described using descriptive statistics. Categorical data are presented as frequencies (percentages) and continuous data as medians (interquartile ranges). All experiments were performed at least in triplicate. Data are expressed as the *m* *e* *a* *n* *s* ± *S* *D*. The Mann–Whitney nonparametric *U* test was utilized to determine differences between the nonmalignant and OC group. When more than two groups were compared (i.e., nonmalignant, early‐stage OC, and advanced‐stage OC), statistical significance was determined using the Kruskal–Wallis test with Dunn′s post hoc correction. *p* < 0.05 was considered significant. Analysis of correlation between variables was assessed using Spearman′s rank correlation coefficient test. To assess the diagnostic potential of biomarkers, the area under the curve (AUC), sensitivity, specificity, and optimal cutoff values of individual biomarkers were determined via ROC curve analysis. All statistical analyses were carried out using the GraphPad Prism 7.04 and SPSS statistical software (SPSS Inc.).

## 3. Results

### 3.1. LD Accumulation in OC Cell Lines and Clinical Tissues

We first evaluated the baseline LD content in various OC cell lines and compared it to normal ovarian (H6036) and HFTEC lines (controls). BODIPY staining revealed a marked increase in LD density in cancer cell lines compared with controls, distinguishing primary cells from cancer cells (Figure 1a). Flow cytometric (Figure 1b) and fluorometric assays (Figure 1c) further confirmed higher LD counts in cancer cell lines than either of the primary cell lines (*p* < 0.001, Student “*t*” test). No significant difference was observed between ovarian (H6036) or HFTEC lines. To corroborate these findings in clinical specimens, we performed BODIPY/DAPI staining on both tumor and normal ovarian tissues, confirming greater LD accumulation in tumor tissues, particularly in advanced‐stage OC, as depicted in Figure 1d.

Figure 1Lipid droplet (LD) accumulation in OC cell lines and ovarian tissue. (a) Cell lines: visualization of LDs using BODIPY 493/503 staining (green) and nuclei staining (blue) in ovarian cancer (OC) cell lines and nonmalignant ovarian (H6036) and HFTEC lines. OC cell lines exhibit a higher quantity of LDs compared with primary cell lines (H6036, HFTEC). Representative images were captured with an inverted microscope (Olympus H4‐100, CCD camera) using a 20× objective. Scale bar represents 100 *μ*m. (b) Cell lines: flow cytometric analysis of BODIPY‐stained cells. Histograms show BODIPY fluorescence intensity, with OC cell lines displaying higher LD content as a peak shift compared with controls (black: H6036, grey: HFTEC). Different OC cell lines are indicated by distinct color peaks (orange: SKOV3, red: OVCAR3, purple: A2780, dark green: A2780‐CDDP, light green: IGROV1, Blue: IGROV1‐CDDP). Data are presented as relative fluorescence intensity in a two‐dimensional FACS profile, with standardized gating for 10,000 events. All experiments were performed in triplicate. (c) Cell lines: quantification of LDs using a microplate fluorometric assay. LD levels in OC cell lines were significantly higher compared with normal control cell line HFTEC. Data represent the mean fold change (FC) of LD content in cancer cell lines relative to normal control cell line (HFTEC) ± SD of triplicate experiments. Statistical significance was determined using Student′s *t*‐test, ∗∗*p* < 0.001 versus control cells. (d) Tissue: visualization of LD content in human ovarian tissue, representing normal, early stage (I) and advanced stage (III) OC samples, using BODIPY staining. Representative images were taken with an inverted microscope (Olympus H4‐100, CCD camera) at 10× magnification.

**Figure:**
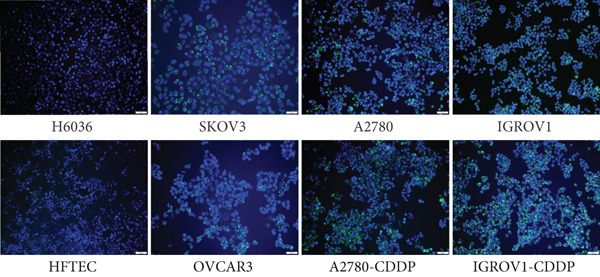
(a)

**Figure:**
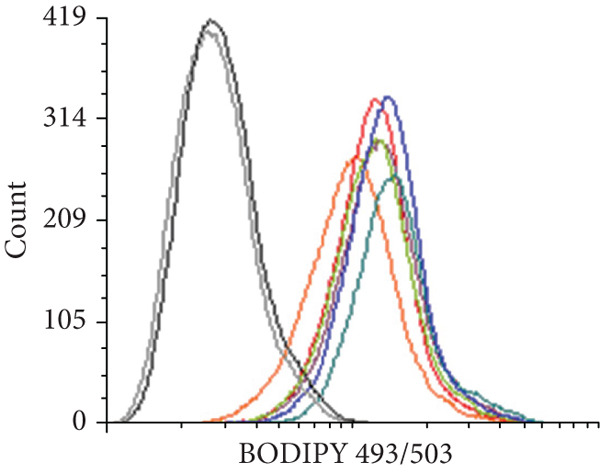
(b)

**Figure:**
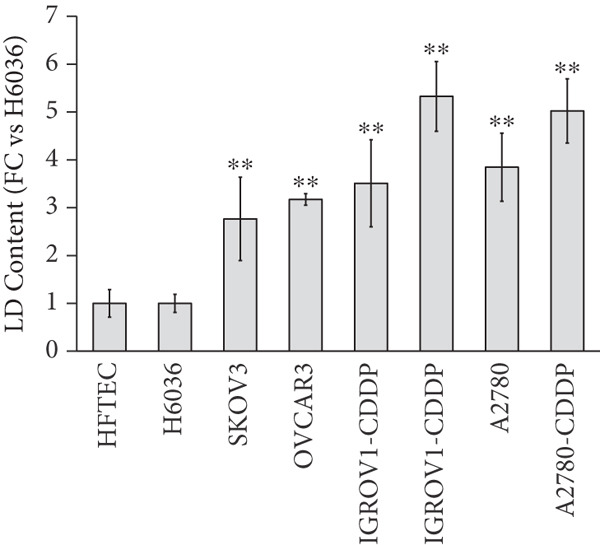
(c)

**Figure:**
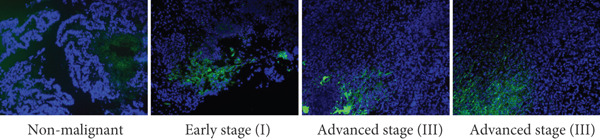
(d)

### 3.2. Increased TG Levels in OC Cell Lines and Clinical Samples

To determine whether TG concentrations accurately reflect the abundance of LDs within cells, we analyzed the correlation between TG levels and LD content. In OC cell lines, TG levels were significantly elevated (two to five times) compared with control HFTEC and H6036 cells (*p* < 0.001; Figure 2a), demonstrating a strong positive correlation with LD content (Spearman *r* = 0.988, *p* < 0.001). No significant TG level differences were observed between ovarian (H6036) and HFTEC lines.

Figure 2Triglyceride (TG) Levels in ovarian cancer. (a) Cell lines: comparison of TG levels between normal control and various ovarian cancer cell lines, quantified using a triglyceride colorimetric assay kit. Values represent mean fold change (FC) ± SD from triplicate experiments. ∗∗*p* < 0.001 compared with normal control cells (Student′s *t*‐test). (b) Tissue: TG levels in ovarian tissue (nonmalignant, *n* = 9; OC patients, *n* = 9; early stage, *n* = 3; advanced stage, *n* = 6) assessed via TG colorimetric assay. Values represent mean *F* *C* ± *S* *D* from triplicate experiments. (c) Tissue: Thin‐layer chromatography analysis of TGs in human ovarian tissue representing normal, early stage (I), and advanced stage (III) ovarian cancer (OC) groups. Lipids were extracted, resolved by thin‐layer chromatography, stained, and quantified. Identification was performed by comparison to standards. Densitometric results of the TLC scan for normal ovary, benign, early stage (I), and advanced (III) stage OC tissue samples are shown on the right. Statistical significance (∗*p* < 0.05) was determined by comparison to normal control cells using the student′s *t*‐test. (d) Peritoneal fluid: TG levels in peritoneal fluid from nonmalignant (*n* = 12) and OC patients (*n* = 20; early stage, *n* = 6; advanced stage, *n* = 14). (e) Plasma: TG levels in plasma from nonmalignant (*n* = 16) and OC patients (*n* = 18; early stage, *n* = 6; advanced stage, *n* = 12). Experiments were performed in triplicate. Box plots display medians (interquartile ranges) and whiskers (minimum and maximum values). Asterisks indicate statistically significant differences compared with the nonmalignant group (∗*p* < 0.05; ∗∗*p* < 0.001; ∗∗∗*p* < 0.0001).

**Figure:**
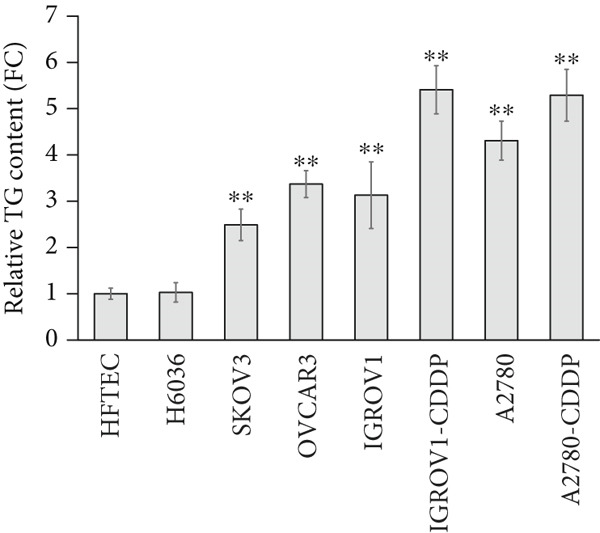
(a)

**Figure:**
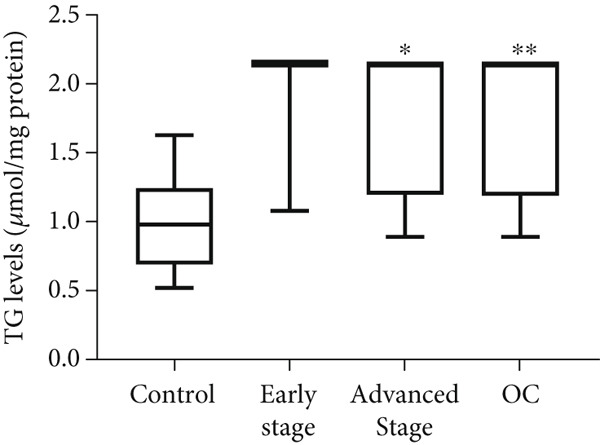
(b)

**Figure:**
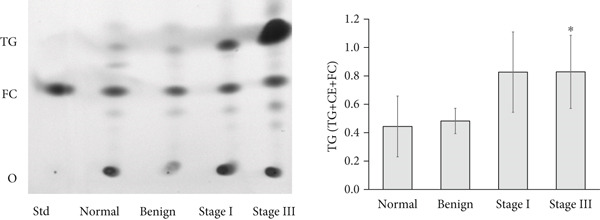
(c)

**Figure:**
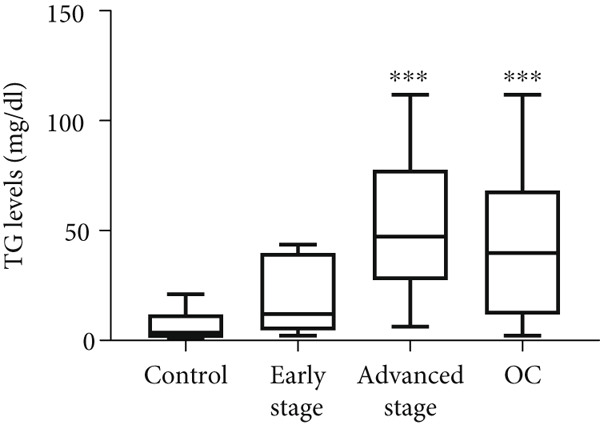
(d)

**Figure:**
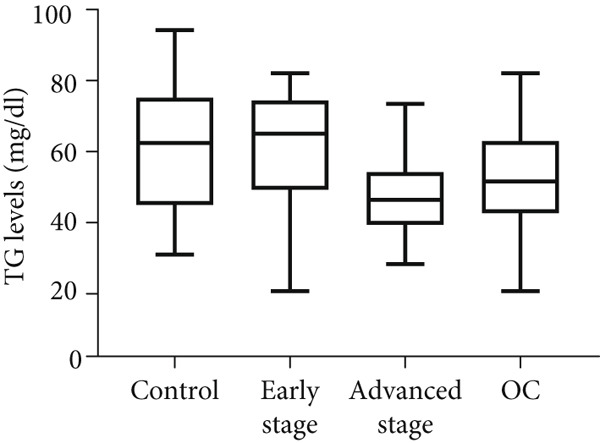
(e)

In tumor tissue, colorimetric assay revealed higher TG levels compared with nonmalignant ovarian tissue (*p* = 0.0056; Figure 2b). The TG levels are significantly higher in the advanced‐stage OC group compared with nonmalignant ovarian tissue (*p* = 0.043; Kruskal–Wallis test with Dunn′s post hoc correction; Figure 2c). The increased TG levels correlated with LD patterns. These findings were further validated by thin‐layer chromatography (TLC). Densitometric analysis revealed higher TG content in tumor tissues, particularly in the advanced‐stage group, compared with normal ovaries or BPM tissues (Figure 2c).

Similar to tumor tissue, TG levels increased significantly in the PF of women with OC compared with the control group (*p* = 0.0001; Figure 2d). Comparing the control group to the early‐ and advanced‐stage OC groups separately, significantly higher levels of TG were observed only in advanced‐stage OC group (*p* = 0.0001; Figure 2d). No significant difference in TG levels was observed between early‐ and advanced‐stage OC groups (*p* > 0.05). Unlike PF and tissue, plasma TG levels did not differ between the OC and control groups (*p* > 0.05; Figure 2e). No significant differences were observed when compared between the control, early‐, and advanced‐stage groups (*p* > 0.05; Figure 2e).

### 3.3. Comprehensive Analysis of DGATs and PLINs in OC Cell Lines and Clinical Samples

We next assessed the expression of DGATs and PLINs gene family in OC cell lines and clinical samples using a diverse methodology, including RT‐qPCR for mRNA expression, ELISA for protein quantification, and bioinformatics to evaluate their diagnostic and prognostic relevance.

### 3.4. OC Cell Lines

Through RT‐qPCR, we demonstrated significantly higher expression of both DGAT1 and DGAT2 mRNA in a panel of OC cell lines compared with primary ovarian epithelial cell line, HFTEC (control, *p* < 0.05; Figure 3a). No significant difference was observed between ovarian (H6036) or HFTEC cell lines. Interestingly, cisplatin‐resistant variants (A2780‐CDDP and IGROV1‐CDDP) of isogenic cell line pairs showed higher DGAT1 and DGAT2 expression compared with their respective cisplatin‐sensitive counterparts (A2780 and IGROV‐1).

Figure 3Analysis of DGAT1 and DGAT2 expression in ovarian cancer. (a) qRT‐PCR analysis of DGAT1 and DGAT2 mRNA expression in various ovarian cancer cell lines compared with primary ovarian epithelial cell line (HFTEC, normal control). The threshold cycle (Ct) values were normalized to the housekeeping gene 18s rRNA using the comparative Ct method to quantify DGATs mRNA levels. Data represent the mean fold change (FC) of mRNA expression in cancer cell lines relative to normal control cell line (HFTEC). Values are shown as mean FC ± SD from triplicate experiments. ∗*p* < 0.05 versus normal control cell line (HFTEC; Student′s *t*‐test). (b) Quantification of DGAT1 and DGAT2 protein levels by ELISA. Data represent the mean FC of protein levels in cancer cell lines relative to normal control cell line (HFTEC). Values are shown as mean FC ± SD from triplicate experiments. ∗*p* < 0.05 versus normal control (Student′s *t*‐test). (c) Western blot analysis of DGAT1 and DGAT2 proteins in various ovarian cell lines, with b‐actin used as the loading control. (d) DGAT1 mRNA transcript levels in ovarian tissue samples (nonmalignant, *n* = 12; OC patients, *n* = 16; early stage, *n* = 4; advanced stage, *n* = 12), assessed via qRT‐PCR. (e) DGAT2 mRNA transcript levels in ovarian tissue samples (nonmalignant, *n* = 12; OC patients, *n* = 16; early stage, *n* = 4; advanced stage, *n* = 12), assessed via qRT‐PCR. Box plots in (d) and (e) display medians (interquartile ranges) and whiskers (minimum and maximum values). Asterisks indicate statistically significant differences compared with the nonmalignant (∗*p* < 0.05; ∗∗*p* < 0.001; ∗∗∗*p* < 0.0001).

**Figure:**
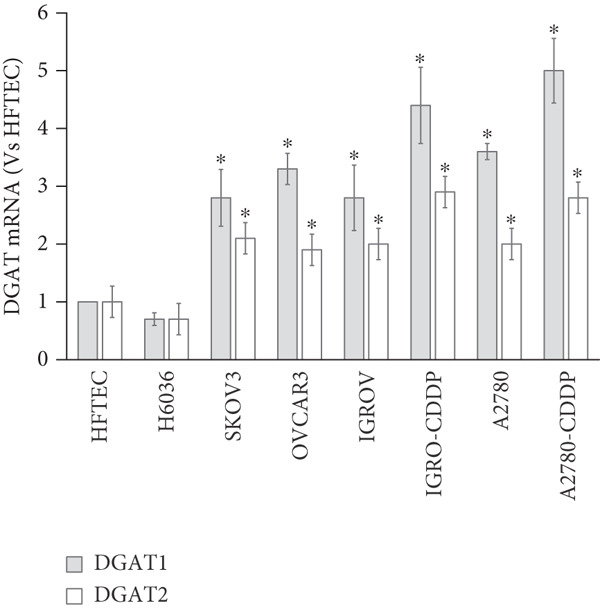
(a)

**Figure:**
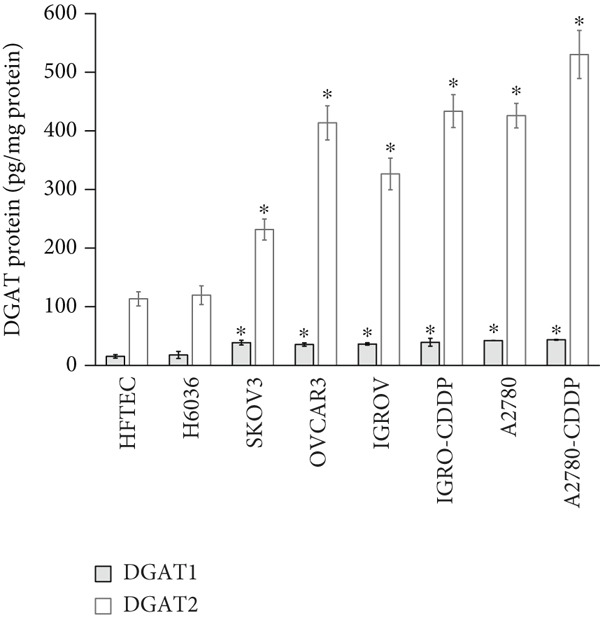
(b)

**Figure:**
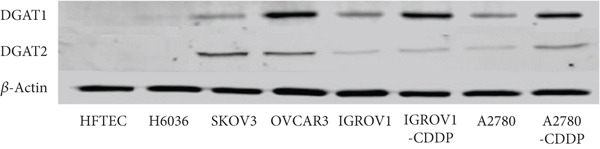
(c)

**Figure:**
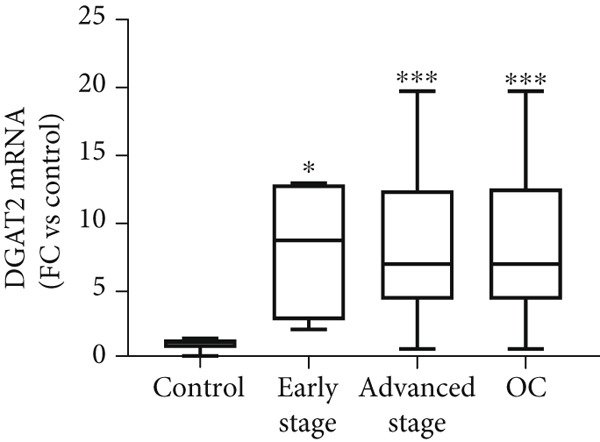
(d)

**Figure:**
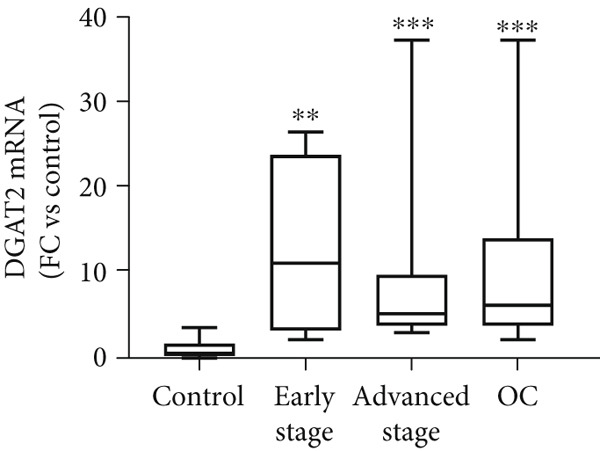
(e)

In exploring protein expression, ELISA results revealed a substantial increase in both DGAT1 and DGAT2 protein levels in OC cell lines compared with control (HFTEC) cell line (Figure 3b). No significant difference was observed between ovarian (H6036) or HFTEC lines. DGAT2 protein levels were significantly higher than DGAT1 in the same cancer cell lines. This finding was corroborated by Western blot analysis (Figure 3c).

Correlation analyses revealed a positive correlation between DGAT1 and DGAT2 mRNA levels in cancer cell lines (Spearman *r* = 0.813, *p* < 0.017), suggesting potential coordinated regulation between these isoforms at the transcriptional level.

### 3.5. Clinical Samples

#### 3.5.1. Tumor Tissue

Analysis of DGAT1 (Figure 3d) and DGAT2 (Figure 3e) expression in ovarian tumor tissues revealed significantly higher mRNA levels compared with nonmalignant ovarian tissue (controls) with statistical significance (*p* < 0.0001 for DGAT1, and *p* < 0.0001 for DGAT2, Mann–Whitney *U* test), indicative of robust transcriptional upregulation in cancer tissues. Upon stratifying the OC group into early OC (Stages I–II) and advanced OC (Stages III–IV), both early‐ and advanced‐stage OC groups exhibited significantly higher DGAT1 and DGAT2 mRNA levels than the control (early: *p* = 0.012 and *p* = 0.0057 for DGAT1 and DGAT2, respectively; advanced: *p* = 0.0009 and *p* = 0.0003 for DGAT1 and DGAT2, respectively, Kruskal–Wallis test with Dunn′s post hoc correction). No significant difference was observed between the early‐ and advanced‐stage OC groups.

Both DGAT1 (Figure 4a) and DGAT2 (Figure 4b) protein levels were significantly higher in OC tissues compared with nonmalignant ovarian tissues (*p* = 0.0449 for DGAT1 and *p* = 0.0121 for DGAT2, Mann–Whitney *U* test). Upon stratification into early‐ and advanced‐ stage OC, a notable increase in DGAT1 and DGAT2 protein levels was observed only in the advanced‐stage group compared with the nonmalignant control group (*p* = 0.0238 and *p* = 0.0351 for DGAT1 and DGAT2, respectively, Kruskal–Wallis test with Dunn′s post hoc correction). No significant difference was observed between early‐ and advanced‐stage OC groups. Interestingly, the increased protein levels in tumor tissues compared with nonmalignant tissues was more pronounced for DGAT1 than for DGAT2, despite DGAT2′s higher baseline levels. This underscores the notable prominence of DGAT1 in the disease context.

Figure 4Quantification of DGAT1 and DGAT2 protein levels in ovarian cancer. (a, b) Tissue: DGAT1 and DGAT2 protein levels quantified by ELISA in levels in ovarian tissue samples (nonmalignant, *n* = 12; OC patients, *n* = 13; early stage, *n* = 4; advanced stage, *n* = 9). (c, d) Peritoneal fluid: DGAT1 and DGAT2 protein levels in peritoneal fluid samples (nonmalignant, *n* = 16; OC patients, *n* = 21; early stage, *n* = 7; advanced stage, *n* = 14). (e, f) Plasma: DGAT1 and DGAT2 protein levels in plasma samples (nonmalignant, *n* = 11; OC patients, *n* = 19; early stage, *n* = 8; advanced stage, *n* = 11). Data represent mean protein concentration ± SD from triplicate experiments. Asterisks indicate statistically significant differences compared with the nonmalignant group (∗*p* < 0.05; ∗∗*p* < 0.001; ∗∗∗*p* < 0.0001).

**Figure:**
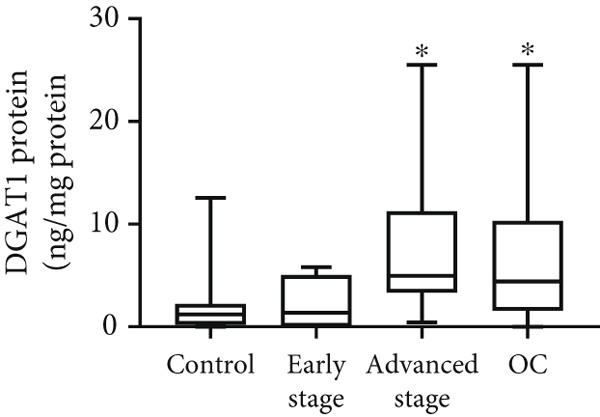
(a)

**Figure:**
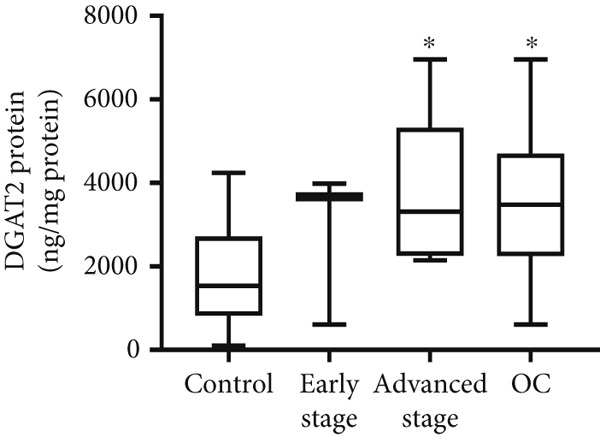
(b)

**Figure:**
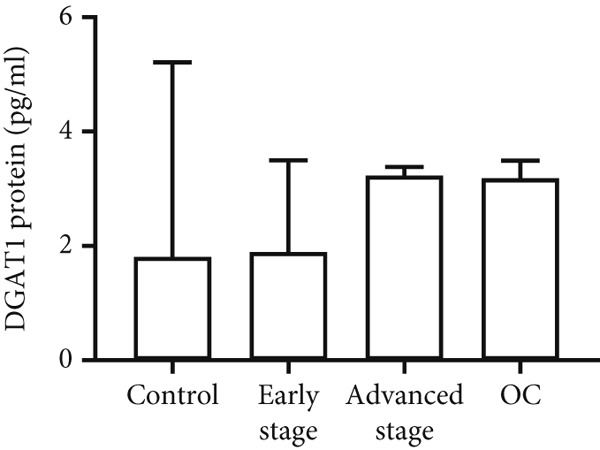
(c)

**Figure:**
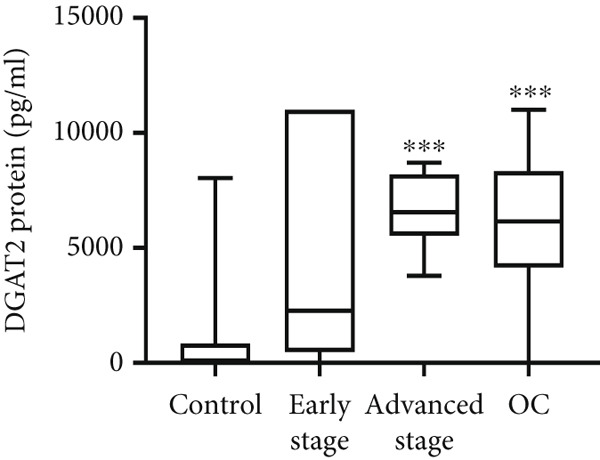
(d)

**Figure:**
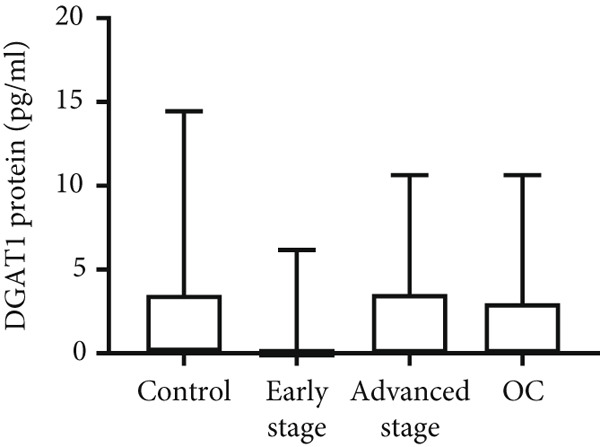
(e)

**Figure:**
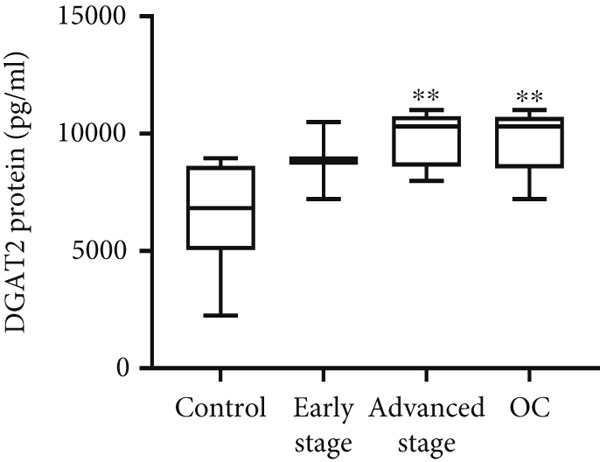
(f)

#### 3.5.2. PF

In PF, DGAT1 protein levels showed no significant difference between nonmalignant controls and OC samples, although a few advanced‐stage cases exhibited higher values that lacked statistical significance (Figure 4c). In contrast, DGAT2 protein levels were significantly elevated in OC samples compared with the nonmalignant group (*p* < 0.0001; Figure 4d). Stratification of OC groups revealed that DGAT2 was significantly higher in the advanced‐stage group compared with nonmalignant controls (*p* < 0.0001), while no difference was observed between early and advanced‐stage groups.

Interestingly, no significant association was observed between DGAT1 and DGAT2 protein levels in PF (Spearman, *p* > 0.05).

#### 3.5.3. Plasma Analysis

Similar to PF, DGAT1 protein levels showed no significant difference between nonmalignant controls and OC samples, with levels generally low or undetectable (Figure 4e). In contrast, DGAT2 protein levels were significantly elevated in OC samples compared with the nonmalignant group (*p* = 0.0013; Figure 4f). Stratification revealed that DGAT2 was significantly higher in the advanced‐stage group compared with nonmalignant controls (*p* = 0.005), whereas no difference was observed between early‐ and advanced‐stage OC groups. Interestingly, no significant association was observed between DGAT1 and DGAT2 proteins in plasma (Spearman, *p* > 0.05).

#### 3.5.4. Correlation of DGATs Protein Levels in Tissue, PF, and Plasma

To determine if PF and plasma DGAT levels can predict tumor DGATs status, we assessed the correlation between PF, plasma, and tissue DGAT protein levels. As shown in Table 2, correlation analyses revealed a significant positive association between tissue DGAT1 and PF DGAT1 protein (Spearman *r* = 0.603, *p* = 0.005). In contrast, no significant association was observed between tissue DGAT1 and plasma DGAT1 protein (Spearman *r* = 0.098, *p* > 0.05) or PF DGAT1 and plasma DGAT1 (Spearman *r* = 0.102, *p* > 0.05). A significant positive association was observed between tissue DGAT2 and PF DGAT2 protein (*r* = 0.571, *p* = 0.029). Interestingly, a significant association was also observed between tissue DGAT2 and plasma DGAT2 (*r* = 0.533, *p* = 0.043). Moreover, a significant association was also observed between PF DGAT2 and plasma DGAT2 protein (Spearman *r* = 0.782, *p* = 0.001).

**Table 2:** Correlations of DGAT protein between tissue, PF, and plasma.

		**Spearman nonparametric analysis**	
		**Spearman** **r** **value**	**p** **value**
DGAT1 protein	Tissue versus PF	0.603	0.005
	Tissue versus plasma	0.098	0.656
	PF versus plasma	0.102	0.643

DGAT2 protein	Tissue versus PF	0.571	0.029
	Tissue versus plasma	0.533	0.043
	PF versus plasma	0.782	0.001

#### 3.5.5. Association Between TG Levels and DGATs

Because DGAT‐dependent TG levels are indicative of LDs, we assessed the correlation between TG levels and DGATs to understand their association with LDs. As shown in Table 3, within tumor tissue, TG levels exhibited a positive correlation with DGAT1 at both the mRNA (*r* = 0.630, *p* = 0.008) and protein levels (*r* = 0.562, *p* = 0.026), while demonstrating a significant positive association with DGAT2 protein (*r* = 0.850, *p* = 0.003). Interestingly, TG levels in PF correlated with tissue DGAT1 mRNA (Spearman *r* = 0.687, *p* = 0.0001) and protein levels (Spearman *r* = 0.533, *p* = 0.013), but not with DGAT2 mRNA (Spearman *r* = 0.361, *p* >0.05) and protein (Spearman *r* = 0.055, *p* > 0.05). This suggests that tissue DGAT1 may play a role in regulating TG secretion into the PF, and the levels of TG in the PF could potentially reflect tissue DGAT1 levels and tumor aggressiveness. Moreover, a positive correlation was detected between PF TG levels and DGAT1 protein (Spearman *r* = 0.552, *p* = 0.010) as well as DGAT2 protein (Spearman *r* = 0.645, *p* = 0.001) levels in the PF. Conversely, TG in plasma did not correlate with any of the isoforms of DGAT in tissue or plasma.

**Table 3:** Correlations analysis between TG and DGATs.

		**Spearman nonparametric analysis**	
		**r** **value**	**p** **value**
Tissue–TG	Versus DGAT1 mRNA (tissue)	0.630	0.008
	Versus DGAT1 protein (tissue)	0.562	0.026
	Versus DGAT2 mRNA (tissue)	0.404	0.109
	Versus DGAT2 protein (tissue)	0.850	0.003

PF–TG	Versus DGAT1 mRNA (tissue)	0.687	0.001
	Versus DGAT1 protein (tissue)	0.533	0.013
	Versus DGAT2 mRNA (tissue)	0.361	0.091
	Versus DGAT2 protein (tissue)	0.055	0.833

Plasma–TG	Versus DGAT1 mRNA (tissue)	−0.100	0.641
	Versus DGAT1 protein (tissue)	−0.256	0.277
	Versus DGAT2 mRNA (tissue)	−0.119	0.573
	Versus DGAT2 protein (tissue)	−0.178	0.481

PF–TG	Versus DGAT1 protein (PF)	0.552	0.010
	Versus DGAT2 protein (PF)	0.645	0.001
	Versus DGAT1 protein (plasma)	−0.188	0.379
	Versus DGAT2 protein (plasma)	0.486	0.041

Plasma–TG	Versus DGAT1 protein (PF)	−0.200	0.384
	Versus DGAT2 protein (PF)	−0.231	0.314
	Versus DGAT1 protein (plasma)	−0.097	0.661
	Versus DGAT2 protein (plasma)	−0.413	0.070

#### 3.5.6. Assessment of Major DGAT Isoform Contributing to LD Levels in OC Cell Lines

Given the significant association between TG and both DGATs in clinical samples, we sought to identify the primary DGAT isoform responsible for the increase in TG and LDs in OC cell lines. Cells were exposed to inhibitors targeting DGAT1 and DGAT2, either individually or in combination, and their LD and TG content was evaluated. As depicted in Figure 5a, both inhibitors effectively decreased LD content across various cell lines. Notably, the DGAT1 inhibitor demonstrated a more significant inhibition of LDs, ranging from 18% to 43%, compared with the DGAT2 inhibitor, which exhibited inhibition ranging from 9% to 24%. The combination treatment demonstrated the highest level of inhibition (ranging from 27% to 64% inhibition) compared with either inhibitor alone. Similarly, DGAT1 inhibition led to a decrease in TG levels (Figure 5b) ranging from 22% to 35%, while DGAT2 inhibition resulted in a decrease of 11% to 23%. The combination treatment led to a more substantial decrease of TG levels (47%–65%). These findings emphasize the contribution of DGATs to LDs via TG synthesis.

Figure 5Effects of DGAT Inhibition on LD and TG levels in ovarian cancer. Ovarian cancer cell lines were treated with DGAT1 inhibitor (PF‐06424439) and DGAT2 inhibitor (A922500), either alone or in combination, for 48 h. (a) LD content was quantified using a microplate fluorometric assay. Media treated cells were considered as control against which treated cells were compared. (b) TG levels was quantified using a microplate colorimetric assay. Media treated cells were considered as control against which treated cells were compared. Data expressed as mean (% inhibition) ± SD of triplicate experiments. Statistical significance at ∗*p* < 0.05, compared with the respective untreated controls. (c) Tissue: Spearman correlation between DGAT1 and Ki67 mRNA in ovarian tissue (*n* = 27). (d) Tissue: Spearman correlation between DGAT2 and Ki67 mRNA in ovarian tissue (*n* = 27).

**Figure:**
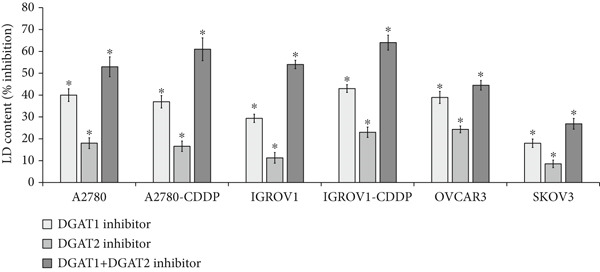
(a)

**Figure:**
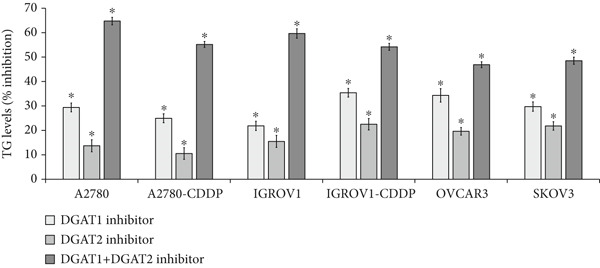
(b)

**Figure:**
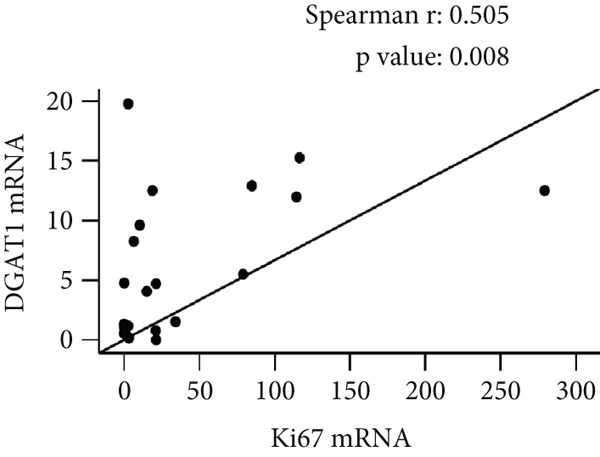
(c)

**Figure:**
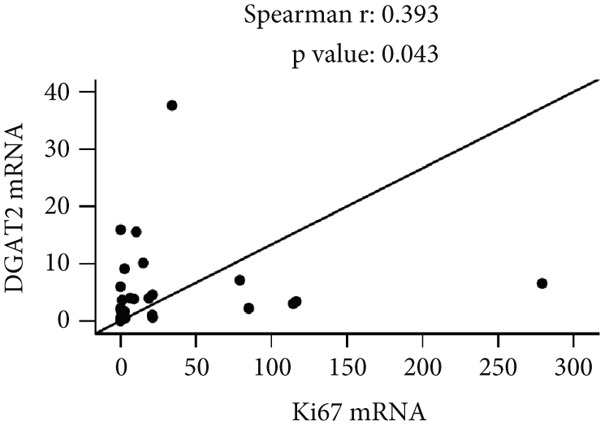
(d)

#### 3.5.7. Correlation Between Ki67 Expression and DGATs (Protein, mRNA)

In our previous investigation, tumor tissue samples from individuals diagnosed with OC exhibited increased levels of Ki67 mRNA transcripts compared with ovarian tissue collected from control subjects [21]. As shown in Figure 5, Spearman correlation analysis revealed significant positive correlations between tissue Ki67 and DGAT1 (Spearman *r* = 0.505, *p* = 0.008; Figure 5c), as well as Ki67 and DGAT2 mRNA transcripts (Spearman *r* = 0.393, *p* = 0.043; Figure 5d).

#### 3.5.8. Analyzing the Expression of PLINs 1‐5 in OC Cell Lines and Tumor Tissue

We determined mRNA expression profiles of PLIN1, PLIN2, PLIN3, PLIN4, and PLIN5 in a panel of OC cell lines and tumor tissue, using RT‐PCR. As shown in Figure 6a, we observed a significant upregulation of PLIN2 and PLIN3 mRNA (*p* < 0.05 and *p* < 0.05, respectively, Mann–Whitney nonparametric *U* test) in a panel OC cell lines compared with primary HFTEC (controls). Conversely, no significant difference was observed in the mRNA levels of PLIN1, PLIN4, and PLIN5 between cancer and control cell lines (*p* > 0.05, Mann–Whitney nonparametric *U* test; Figure 6b). These findings were further confirmed in tumor tissues. Consistent with the cell line results, no significant difference was observed in the expression of PLIN1 (Figure 6c) between tumor and nonmalignant ovarian tissue (*p* > 0.05, Mann–Whitney nonparametric *U* test). Significant upregulation of PLIN2 (*p* = 0.0048, Mann–Whitney nonparametric *U* test; Figure 6d) and PLIN3 mRNA (*p* = 0.0041, Mann–Whitney nonparametric *U* test; Figure 6e) in OC tissue compared with nonmalignant ovarian tissue. Upon stratification of the OC group into early and advanced stages, only the advanced‐stage group exhibited significantly higher PLIN2 mRNA levels compared with the nonmalignant control (*p* = 0.031; Kruskal–Wallis test with Dunn′s post hoc correction; Figure 6d). No significant difference was observed between the early‐ and advanced‐stage OC groups. Interestingly, PLIN3 mRNA levels were significantly higher in the early‐stage group compared with controls (*p* = 0.047; Kruskal–Wallis test with Dunn′s post hoc correction; Figure 6e). There were no significant differences in the expression of PLIN4 (Figure 6f) and PLIN5 (Figure 6g) between tumor and nonmalignant ovarian tissue (*p* > 0.05, Mann–Whitney nonparametric *U* test).

Figure 6qRT‐PCR analysis of PLINs 1–5 mRNA expression in ovarian cancer. To quantify mRNA, the threshold cycle (Ct) values were normalized to the housekeeping gene 18s rRNA using the comparative Ct method. Data represent the mean fold change (FC) of mRNA expression in cancer cell lines or tissues relative to their respective normal controls, shown as mean *F* *C* ± *S* *D* from triplicate experiments, significant at ∗*p* < 0.05 versus normal/nonmalignant controls. qRT‐PCR analysis of PLIN1 and PLIN2 (a) and PLIN3, PLIN4, and PLIN5 mRNA expression (b) in a panel of ovarian cancer cell lines compared with primary fallopian epithelial cell (HFTEC) line (normal control). Significant at ∗*p* < 0.05, Student “*t*” test. (c–g) Tissue: PLIN mRNA transcript levels in ovarian tissue samples, assessed via qRT‐PCR (nonmalignant, *n* = 11; *O* *C* = 13; *e* *a* *r* *l* *y* *s* *t* *a* *g* *e* = 4, *a* *d* *v* *a* *n* *c* *e* *d* *s* *t* *a* *g* *e* = 9). (c) Tissue: PLIN1 mRNA levels in nonmalignant (*n* = 19) and OC groups. Tissue: (d) PLIN2 mRNA levels, (e) PLIN3 mRNA levels, (f) PLIN4 mRNA levels, and (g) PLIN5 mRNA levels. Box plots show medians (interquartile ranges) and whiskers (minimum and maximum values). Asterisks indicate statistically significant differences compared with nonmalignant controls (∗*p* < 0.05; ∗∗*p* < 0.001).

**Figure:**
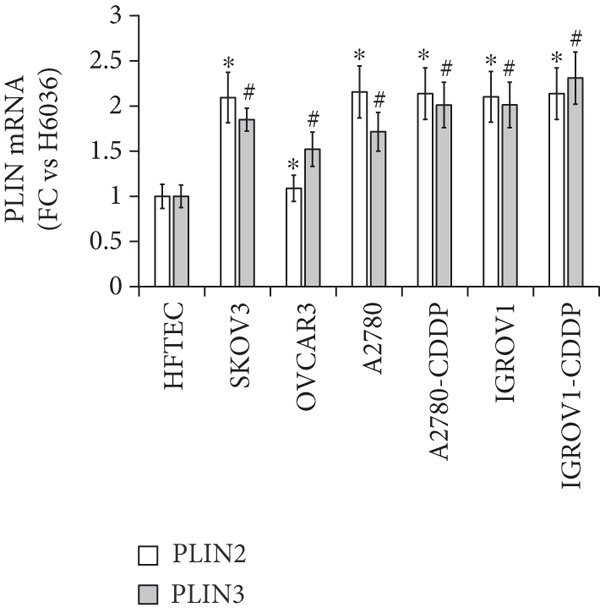
(a)

**Figure:**
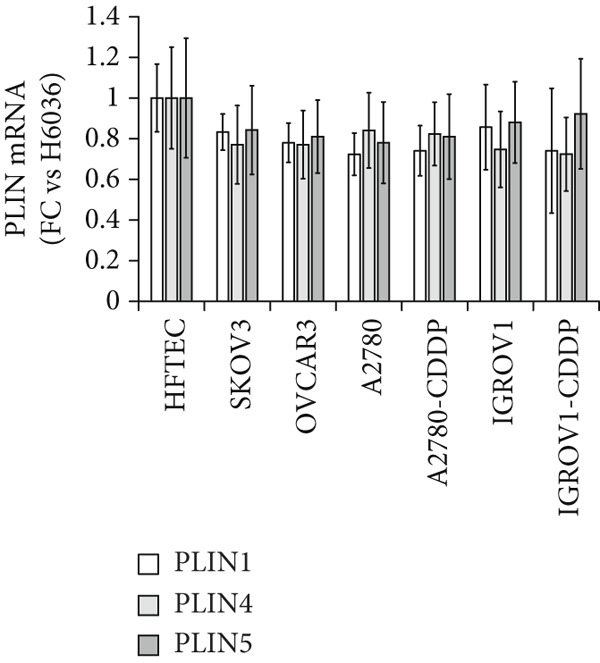
(b)

**Figure:**
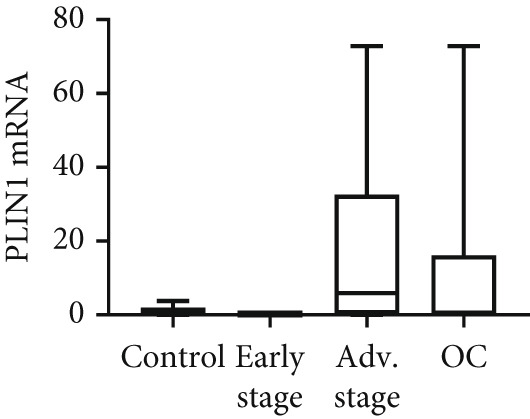
(c)

**Figure:**
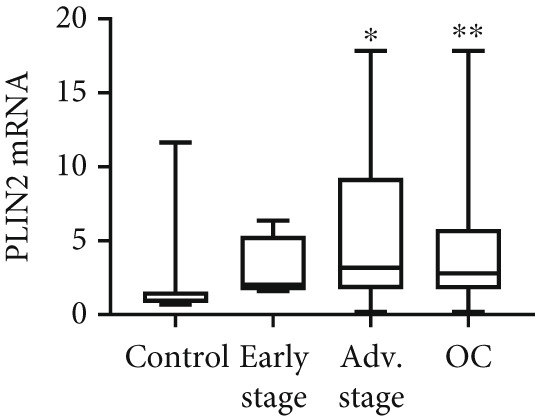
(d)

**Figure:**
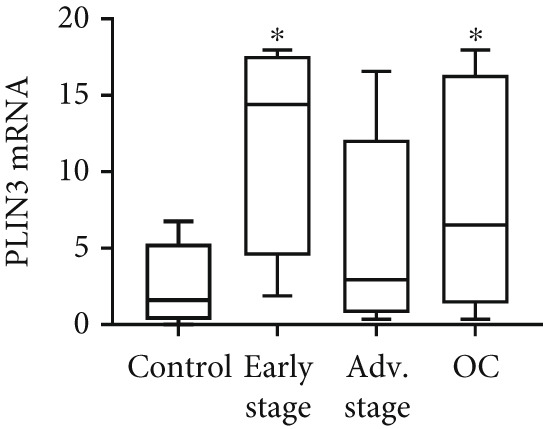
(e)

**Figure:**
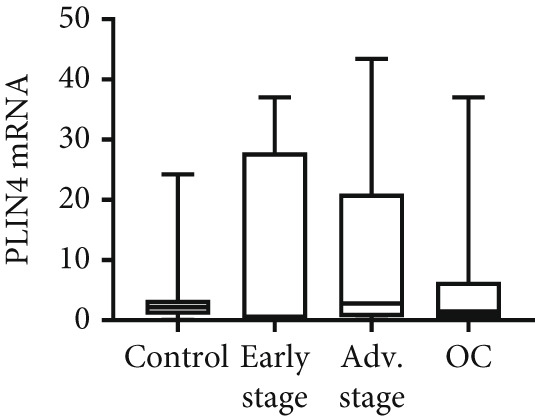
(f)

**Figure:**
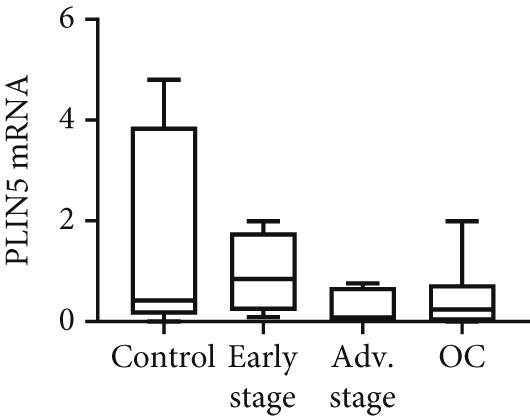
(g)

### 3.6. Bioinformatics Analysis

#### 3.6.1. Expression and Genetic Alterations of LD‐Associated Genes in OC

We utilized the GEPIA2 platform to validate the expression of DGATs and PLINs at both mRNA and protein levels in OC and matched normal tissues (Figure S1). While DGAT1 mRNA levels were higher in the OC group compared with the normal group, the difference did not reach statistical significance (Figure S1a). Consistent with our findings, statistical analysis revealed a significant upregulation of DGAT2 expression in the OC group compared with the normal group (Figure S1b). In contrast to our findings, GEPIA2 analysis revealed significant differences in PLIN1 (Figure S1c) and PLIN5 (Figure S1g) expression between the OC group and the control group (*p* < 0.0001). However, PLIN2 (Figure S1d), PLIN3 (Figure S1e), and PLIN4 (Figure S1f) did not show significant differences between the OC and control groups.

Furthermore, we utilized cBioPortal cancer databases to investigate genetic alterations in these genes (Figure S2). The genetic alteration data from 1880 patients revealed that 28% of OC patients exhibited alterations in the DGAT1 gene, 8% in DGAT2, 4% in PLIN1, 3% in PLIN2, and 2% each in PLIN3, PLIN4, and PLIN5 genes. Specifically, genetic alterations in DGAT1, DGAT2, PLIN1, and PLIN2 were predominantly characterized by copy number amplifications, while deep deletions were observed for PLIN4 and PLIN5 genes.

#### 3.6.2. Prognostic Evaluation of Key LD‐Associated Genes in OC

Survival analysis could not be conducted in our study due to the limited follow‐up time and a small sample size. Nonetheless, we employed Kaplan–Meier plotter and cBioportal to evaluate the prognostic significance of these genes in OC. As shown in Figure 7, comparing survival curves between the groups based on their median expression levels, DGAT1 has no prognostic potential (Figure 7a). Interestingly, analysis from cBioportal indicated that individuals with altered DGAT1 expression survived significantly longer than those with unaltered DGAT1 expression (*H* *R* = 0.804, 95% CI: 0.701–0.922, log‐rank *p* = 0.002708; Figure S3a), suggesting DGAT1 expression as a potential favorable prognostic marker for OC. Similarly, individuals with high DGAT2 and PLIN2 expression exhibited significantly lower survival rates than those with low expression levels of DGAT2 (*H* *R* = 1.39, 95% CI: 1.14–1.71, log‐rank *p* = 0.0012; Figure 7b) and PLIN2 (*H* *R* = 1.23, 95% CI: 1.08–1.4, log‐rank *p* = 0.0015; Figure 7c). This suggests that DGAT2 and PLIN2 expression may serve as potential unfavorable prognostic markers for OC. Conversely, individuals with high PLIN3 expression demonstrated significantly higher survival rates than those with low PLIN3 expression (*H* *R* = 0.84, 95% CI: 0.73–0.96, log‐rank *p* = 0.013; Figure 7d). As shown in Figure S3, no correlation was observed between the mRNA expression of PLIN1 (Figure S3b), PLIN4 (Figure S3c), and PLIN5 (Figure S3b) with OS in OC. Additional bioinformatics analyses are required to validate the efficacy of LD‐associated genes and to develop prognostic algorithms.

Figure 7Prognostic significance of DGAT1, DGAT2, PLIN2, and PLIN3 expression in ovarian cancer. Kaplan–Meier survival curves showing overall survival based on high and low expression of (a) DGAT1, (b) DGAT2, (c) PLIN2, and (d) PLIN3, generated using the Kaplan–Meier Plotter database.

**Figure:**
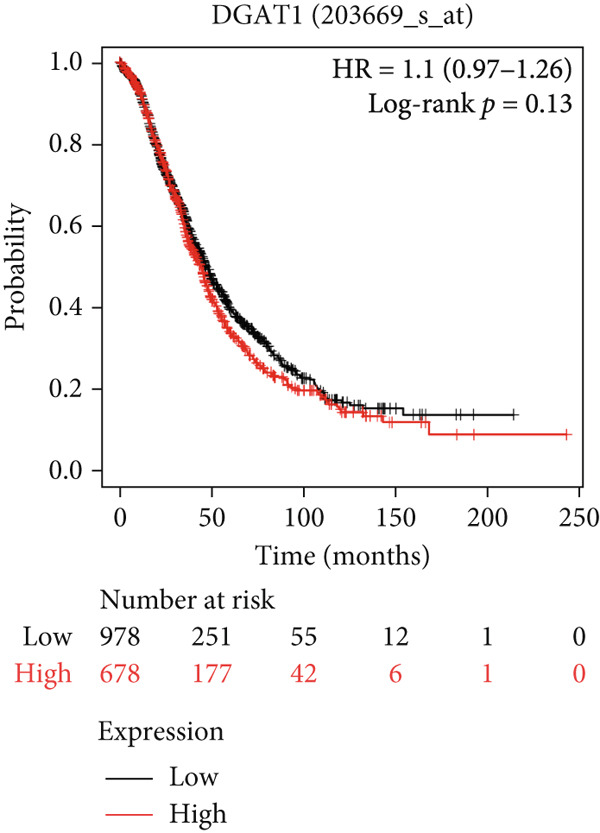
(a)

**Figure:**
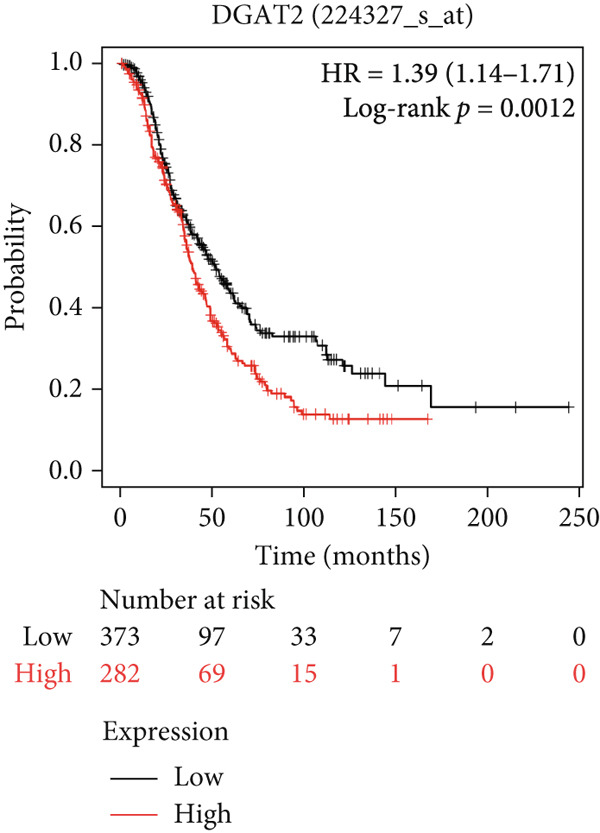
(b)

**Figure:**
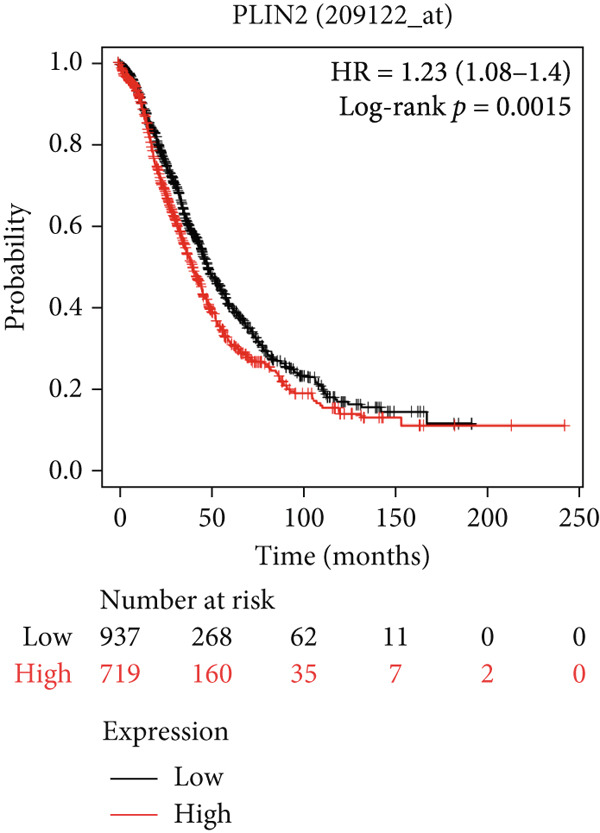
(c)

**Figure:**
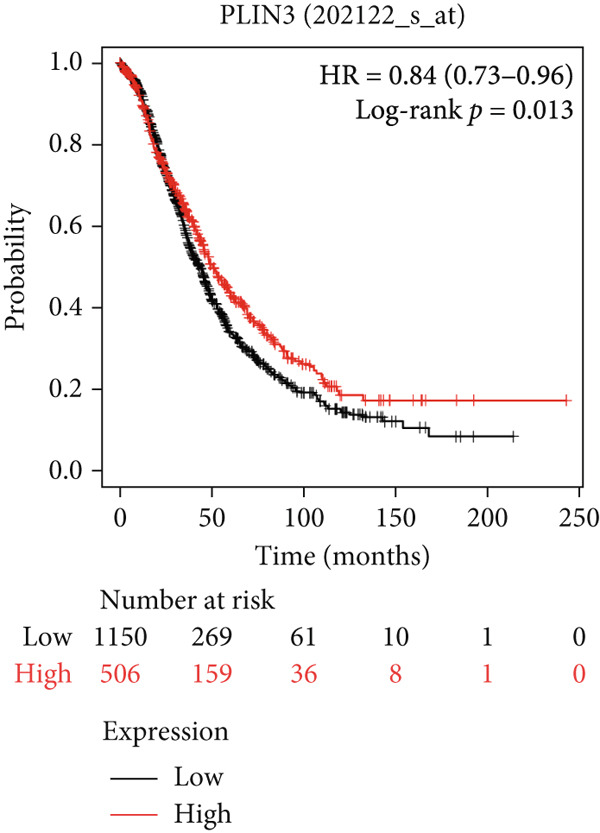
(d)

#### 3.6.3. Evaluation of DGATs and TG as Diagnostic Indicators of OC

The diagnostic capabilities of DGAT1, DGAT2, and TG in plasma, PF, and tissue were assessed using ROC curve analysis for diagnosing OC. As shown in Table 4, evaluating DGAT2 levels in PF (*A* *U* *C* = −0.8869), plasma (*A* *U* *C* = −0.9038), and OC tissue (*A* *U* *C* = −0.9844) demonstrated superior diagnostic efficacy compared with DGAT1. While DGAT1 in tissue exhibited good diagnostic potential with an AUC of 0.95, its detection in PF and plasma was minimal. Similarly, TG levels in tissue and PF displayed sound diagnostic potential with AUC values of 0.82 and 0.89, respectively, but plasma TG levels did not correlate with malignancy. Intriguingly, mRNA levels of PLIN2 and PLIN3 were able to differentiate between malignant and nonmalignant tissue with AUC values of 0.8322 and 0.7483, respectively.

**Table 4:** LD‐associated genes and TG as diagnostic indicators for OC.

	**Analyte**	**AUC value**	**p** **value**
Tissue	DGAT1‐mRNA	0.9479	<0.0001
	DGAT2‐mRNA	0.9844	<0.0001
	DGAT1‐protein	0.7413	0.0456
	DGAT2‐protein	0.7564	0.0296

PF	DGAT1‐protein	0.5286	0.8148
	DGAT2‐protein	0.8869	<0.0001

Plasma	DGAT1‐protein	0.622	0.2725
	DGAT2‐protein	0.9038	0.0024

Tissue	TG/(TG + FS + CE)	0.8167	0.02
PF	TG	0.8854	0.0003
Plasma	TG	0.6528	0.129

Tissue	PLIN1‐mRNA	0.5315	0.7943
	PLIN2‐mRNA	0.8322	0.0059
	PLIN3‐mRNA	0.7483	0.0397
	PLIN4‐mRNA	0.5175	0.8848
	PLIN5‐mRNA	0.6573	0.1924

## 4. Discussion

Current treatments for OC are frequently undermined by the development of chemoresistance and high recurrence rates [1, 24], emphasizing the critical need for novel biomarkers to improve patient outcomes. Our study has explored the therapeutic, prognostic, and diagnostic implications of LD‐associated genes in OC. The accumulation of LDs was associated with elevated levels of TGs, DGATs, and PLINs (PLIN1–PLIN5) in OC, suggesting their roles in LD synthesis and LD formation. We comprehensively analyzed the expression of these LD‐associated genes in both OC cell lines and clinical samples. Our results revealed significantly higher expression levels of DGAT1, DGAT2, TG, and PLIN2 proteins in OC compared with normal controls across cell lines and clinical specimens, indicating the crucial involvement of lipid storage mechanisms in OC pathophysiology. Notably, we found significant correlations between DGAT1 and DGAT2 mRNA expression and the tumor aggressiveness marker Ki67. Furthermore, a significant association was found between elevated levels of DGAT1, DGAT2, PLIN2, PLIN3, and TG and malignancy, especially in advanced tumor stages, underscoring their promise as therapeutic targets in OC.

Bioinformatic survival analysis demonstrated that high expression of DGAT2 and PLIN2 correlated with decreased OS in OC, while elevated PLIN3 expression was associated with enhanced OS. Diagnostic assessments using ROC analysis revealed that elevated DGAT2 levels detected in tumor tissue, PF, and plasma exhibited robust diagnostic capabilities. Moreover, significant Spearman correlations between DGAT2 expression in tumor tissue and plasma, and between PF and plasma, indicate that plasma DGAT2 reliably reflects tumor‐associated expression. These findings support DGAT2 as a noninvasive biomarker for OC, with plasma measurement serving as a surrogate for tumor burden and minimizing the need for invasive sampling. Additionally, DGAT1, TGs, PLIN2, and PLIN3 also showed considerable diagnostic potential for detecting malignancy in tissue.

The fact that altered lipid metabolism is observed in all stages of OC suggests a significant association between OC and lipids [3, 25, 26]. OC cells acquire large amounts of lipids to meet their energy, structural, and membrane signaling needs [3]. Intra‐abdominal tumors, including OC, tend to metastasize preferentially to the omentum, a tissue predominantly consisting of adipocytes [3, 27]. Excessive lipids within non‐cancerous cells promote lipotoxicity and inhibit growth factor signaling, leading to cellular stress; however, cancer cells are uniquely equipped to mitigate these effects [6, 28]. The biogenesis of LDs is a critical adaptive mechanism in cancer cells, allowing them to store excess lipids and avoid lipotoxicity [29–32]. These LDs play multifunctional roles in stressed cells, such as sequestering potentially toxic lipids, maintaining energy and redox balance, regulating FA trafficking, autophagy, and enhancing chemoresistance [4–8]. Given their diverse protective functions and accumulation in OC tissues, LDs represent novel therapeutic targets for OC. However, the specific molecular mechanisms underlying LD accumulation and their precise functions within OC remain poorly understood. Identifying and targeting these mechanisms could unveil new therapeutic avenues for cancer treatment.

DGAT enzymes, particularly DGAT1 and DGAT2, are central to the metabolic reprogramming observed in tumors. These enzymes, responsible for TG synthesis and LD formation, are often overexpressed in various cancers and are linked to enhanced tumor growth, aggressiveness, and chemoresistance, rendering them attractive targets for intervention [15–18, 33–35]. DGAT1, primarily expressed in the small intestine and duodenum, is involved in TG storage, whereas DGAT2, found in adipocytes and the liver, regulates systemic TG levels and postnatal survival [33]. Existing research consistently highlights the overexpression of DGAT1 and DGAT2 in various cancers, including prostate [17, 18], breast [5, 33, 36], and OCs [19]. Genetic analyses through platforms like cBioPortal indicate varying genetic alterations in these enzymes across different cancers.

While DGAT1 inhibition suppresses tumor growth in various cancers, including glioblastoma [15], melanoma [28], and prostate cancer [17, 18], overexpression of DGAT2 inhibits cell proliferation in hepatocellular carcinoma (HCC) [37, 38]. Additionally, DGAT1 and DGAT2 play crucial roles in tumor dynamics by modulating epithelial‐mesenchymal transition (EMT), influencing both tumor invasion and metastasis [34]. Inhibitors of these enzymes have demonstrated effectiveness in preclinical studies by disrupting cancer cell metabolism and reducing tumor growth across various cancer types. In prostate cancer cells, the knockdown of DGAT1 leads to decreased cell growth and increased autophagy, suggesting a potential therapeutic target [17, 18]. In breast cancer, overexpression of the proto‐oncogene HER2 leads to lipogenic reprogramming, including upregulation of DGAT enzymes, further implicating their involvement in oncogenic processes [33, 36]. Expanding on the role of DGAT enzymes in cancer progression, the accumulation of LDs driven by DGAT activity is crucial in chemoresistance, as LDs sequester lipophilic drugs, substantially reducing chemotherapy effectiveness [33, 34]. Therefore, targeting DGAT enzymes may improve therapeutic outcomes.

The clinical relevance of DGATs in prognosis varies significantly across different cancer types. Overexpression of DGAT1 is associated with poorer survival outcomes in OC and gastric cancer, whereas it correlates with improved survival in lung adenocarcinoma [15, 16, 34]. Conversely, higher levels of DGAT2 are linked to extended survival in patients with HCC [37, 39]. This variability underscores the complexity of DGAT enzymes as prognostic targets, emphasizing the need for cancer‐specific studies to fully understand their roles in cancer metabolism and progression. Additionally, understanding the mechanisms underlying DGAT regulation and their impact on lipid metabolism is crucial for elucidating their therapeutic and prognostic significance. DGAT1 and DGAT2 are promising targets in cancer treatment, playing critical roles in LD formation and tumor progression, highlighting their potential for developing new therapeutic strategies. Further research into the regulation of DGATs and their effects on lipid metabolism across different cancer types will deepen our understanding of their clinical significance and facilitate the development of targeted therapies to enhance patient outcomes.

PLINs are crucial proteins that regulate LDs in cells, impacting cancer cell metabolism, growth, survival, and therapy response [8–10]. Covering the LD surface, PLINs play a central role in controlling lipolysis, the breakdown of lipids into FAs and glycerol. The five primary PLIN proteins—PLIN1 through PLIN5—have distinct functions influenced by their expression patterns and tissue types. PLIN1, PLIN2, and PLIN5 may modulate lipid metabolism by interacting with lipolytic enzymes or hindering their access to LDs, while PLIN3 and PLIN4 may be involved in intracellular neutral lipid packaging and trafficking.

PLIN1 is a prominent structural protein found abundantly on LDs and is highly expressed in mature adipocytes. Recent research indicates that the absence of PLIN1 reduces LD formation by downregulating nuclear SREBP‐1, likely through interactions with cytosolic lipases, thereby affecting lipolysis [40, 41]. Conversely, PLIN2 exhibits ubiquitous distribution, with significant expression in the liver, where its levels positively correlate with TG levels and LD formation [42, 43]. It regulates adipocyte differentiation and LD accumulation under PPARs and RXR control [44, 45]. During adipogenic differentiation, PLIN2 is replaced by PLIN1, potentially hindering lipolytic pathways by interfering with the association of ATGL with LDs, consequently increasing TAG levels and LD accumulation [46]. Overexpression of PLIN2 has been reported in various tumors, including Burkitt lymphoma [47], malignant melanoma [48], renal cell carcinoma [49], lung adenocarcinoma [50], and breast cancer [51].

PLIN3, similar to PLIN2, exhibits widespread expression and is implicated in LD formation and prostaglandin E2 (PGE2) generation [52]. Upon exposure to excess FAs, PLIN3 translocates from the cytoplasm to nascent LDs, facilitating TG biosynthesis and storage. Elevated PLIN3 levels have been observed in multiple malignancies, including HCC, breast cancer, colon cancer, and lung cancer [53–55]. However, the correlation between PLIN3 expression and patient prognosis varies among different cancer types [13, 56]. PLIN4 and PLIN5 also have distinctive functions. PLIN4 appears late in adipocyte differentiation and, along with PLIN3 and PLIN2, preferentially binds to CE‐enriched LDs upon adipogenic stimulation [57]. Meanwhile, PLIN5, predominantly present in organs specializing in FA oxidation such as the heart and liver, translocates to LDs during lipogenic processes [58, 59]. It aids in counteracting the detrimental effects of excessive FA oxidation.

LD‐associated genes, notably DGATs and PLINs, significantly contribute to tumor development and prognosis across various cancers. They orchestrate LD formation within tumor tissues via intricate pathways linked to inflammation, hypoxia, and acidosis, intersecting with key oncogenic pathways like PTEN, KRAS, and FOXO3/Sirtuin6 [5, 43]. In OC, these genes play crucial roles in AMPK/ACC/FASN‐mediated lipogenesis and AMPK/TAK1/NF‐*κ*B signaling, rendering them promising targets for innovative therapeutic approaches [60, 61]. Ongoing research aims to unveil the specific functions and mechanisms of DGATs and PLINs in cancer, potentially revolutionizing oncology diagnostics and treatments.

## 5. Conclusions

LD significantly influence tumor growth, facilitating chemoresistance and disease recurrence. Consequently, LDs and associated genes, such as DGATs and PLINs, are important targets for cancer therapy. Ongoing research in these fields is broadening horizons for diagnostic, prognostic and therapeutic strategies in OC, highlighting the value of metabolic enzymes as both biomarkers and therapeutic targets. This approach offers promising prospects for the development of targeted cancer therapies aimed at substantially improving patient survival and quality of life.

**Nomenclature**
DGAT1: diacylglycerol O‐acyltransferase 1
DGAT2: diacylglycerol O‐acyltransferase 2
TG: triglycerides
OC: ovarian cancer
LD: lipid droplets
ROC: receiver operating characteristic
qRT‐PCR: quantitative real‐time polymerase chain reaction
TLC: thin‐layer chromatography
BMI: body mass index
AUC: area under the curve
IGROV‐1CDDP/A2780‐CDDP: cisplatin‐resistant variants of IGROV‐1 and A2780

## Disclosure

All authors have read and approved the final manuscript.

## Conflicts of Interest

The authors declare no conflicts of interest.

## Author Contributions

Conceptualization: V.N.A. and L.B.; data curation: V.N.A., M.L., Z.P., and P.D‐S.; formal analysis: V.N.A., M.L., P.D‐S., and K.G.; funding acquisition: L.B.; investigation: V.N.A., M.L., and L.B.; methodology: V.N.A., M.L., Z.P., P.D‐S., K.G., and L.B.; project administration: V.N.A., P.D‐S., K.G., T.W., and L.B.; resources: L.B.; software: V.N.A., K.G., and P.D‐S.; supervision: V.N.A. and L.B.; validation: V.N.A., M.L., P.D‐S., and L.B.; visualization: V.N.A., P.D‐S., and K.G.; writing (original draft): V.N.A., P.D‐S., K.G., and L.B.; writing (review and editing): V.N.A., P.D‐S., K.G., and L.B.

## Funding

The authors received no specific funding for this work.

## Supporting information


**Supporting Information** Additional supporting information can be found online in the Supporting Information section. Table S1: Primers for RT‐qPCR targeting DGATs and PLINs. Figure S1: GEPIA2 analysis of key lipid droplet‐associated genes. The mRNA expression levels of DGAT1 (a), DGAT2 (b), PLIN1 (c), PLIN2 (d), PLIN3 (e), PLIN4 (f), and PLIN5 (g) in ovarian cancer were analyzed using the GEPIA2 platform. The expression levels of these LD‐associated genes were compared between ovarian cancer tissue (T) and normal ovarian tissue (N). The differentially expressed genes are presented in bar plots, highlighting differences in mRNA expression levels between ovarian cancer and normal ovarian tissue. ∗Significant at *p* < 0.05. Figure S2: DGATs oncoprint. cBioPortal “oncoprint” representation of alterations in DGATs and PLIN genes identified in ovarian cancer consisting of 1937 samples (amplification, red; deep deletion, blue; no alterations, grey). Numbers represent the combined frequency of all alterations. TCGA datasets selected are shown above. Figure S3: Survival curves of LD genes. Prognostic value of the PLIN1 (a), PLIN4 (b), and PLIN5 (c) and genes in ovarian cancer patients using the Kaplan–Meier plotter online database. (d) Prognostic value of the DGAT1 gene, comparing DGAT1 altered versus unaltered groups in ovarian cancer, using cBioportal database.

## Data Availability

The data that support the findings of this study are available from the corresponding author upon reasonable request.
