# Supplementary material for: Comprehensive Analysis of DGATs and PLINs in Ovarian Cancer: Implications for Diagnosis and Prognosis
Source: Biomed Res Int. 2025 Oct 1;2025:9153643. doi: 10.1155/bmri/9153643 (PMC12487632; doi:10.1155/bmri/9153643)

Supplementary Table 1: Primer for RT-qPCR targeting DGATs and PLINs

| Primer | Forward 5'-3' | Reverse 5-3' |
| --- | --- | --- |
| DGAT1 | GCT TCA GCA ACT ACC GTG GCA T | CCT TCA GGA ACA GAG AAA CCA CC |
| DGAT2 | CTA CAG GTC ATC TCA GTG CTC | CAC CAG CCA AGT GAA GTA GA |
| PLIN1 | CTT TAA CCA AAC TTG TGG CC | TAC TCA GAA AGT GAC ACT AG |
| PLIN2 | AGT GGA AAA GGA GCA TTG GA | GTC TCC TGG CTG CTC TTG TC |
| PLIN3 | GCT ACT TCG TAC GTC TGG GGC | TTT CTC AGT GAT TCC AGG GG |
| PLIN4 | CCA AAG ACC TGG TGT GTT CC | AGC ACA GCC TTG GAG GTT T |
| PLIN5 | GTG GCC AGC AGT GTC ACG GG | GGA GCC GAG GCG CAC AAA GT |
| RPL4 | GCTCTGGCCAGGGTGCCTTTG | ATGGCGTATCGTTTTTGGGTTGT |
| beta-Actin | AGCACTGTGTTGGCGTACAG | GGACTTCGAGCAAGAGATGG |


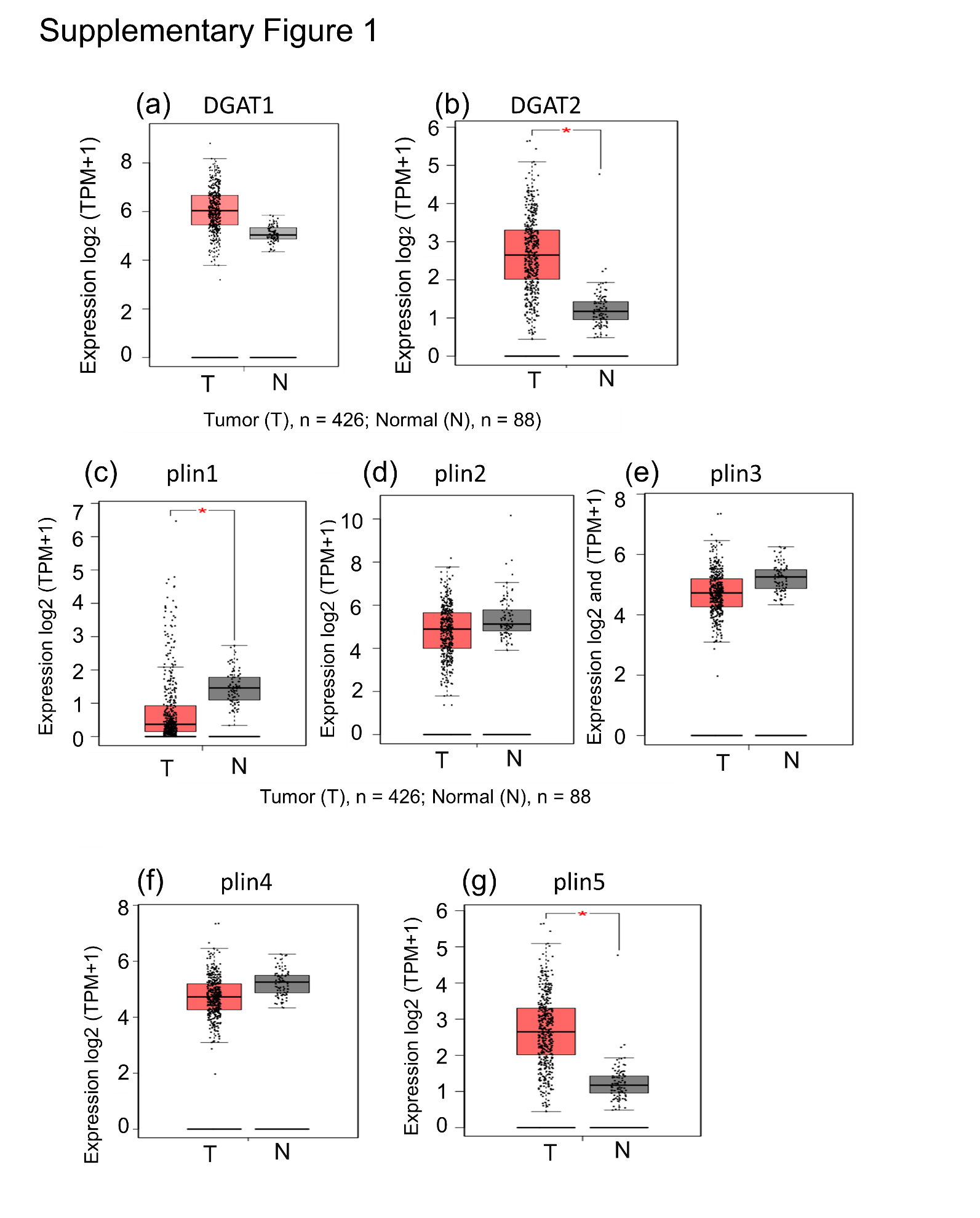


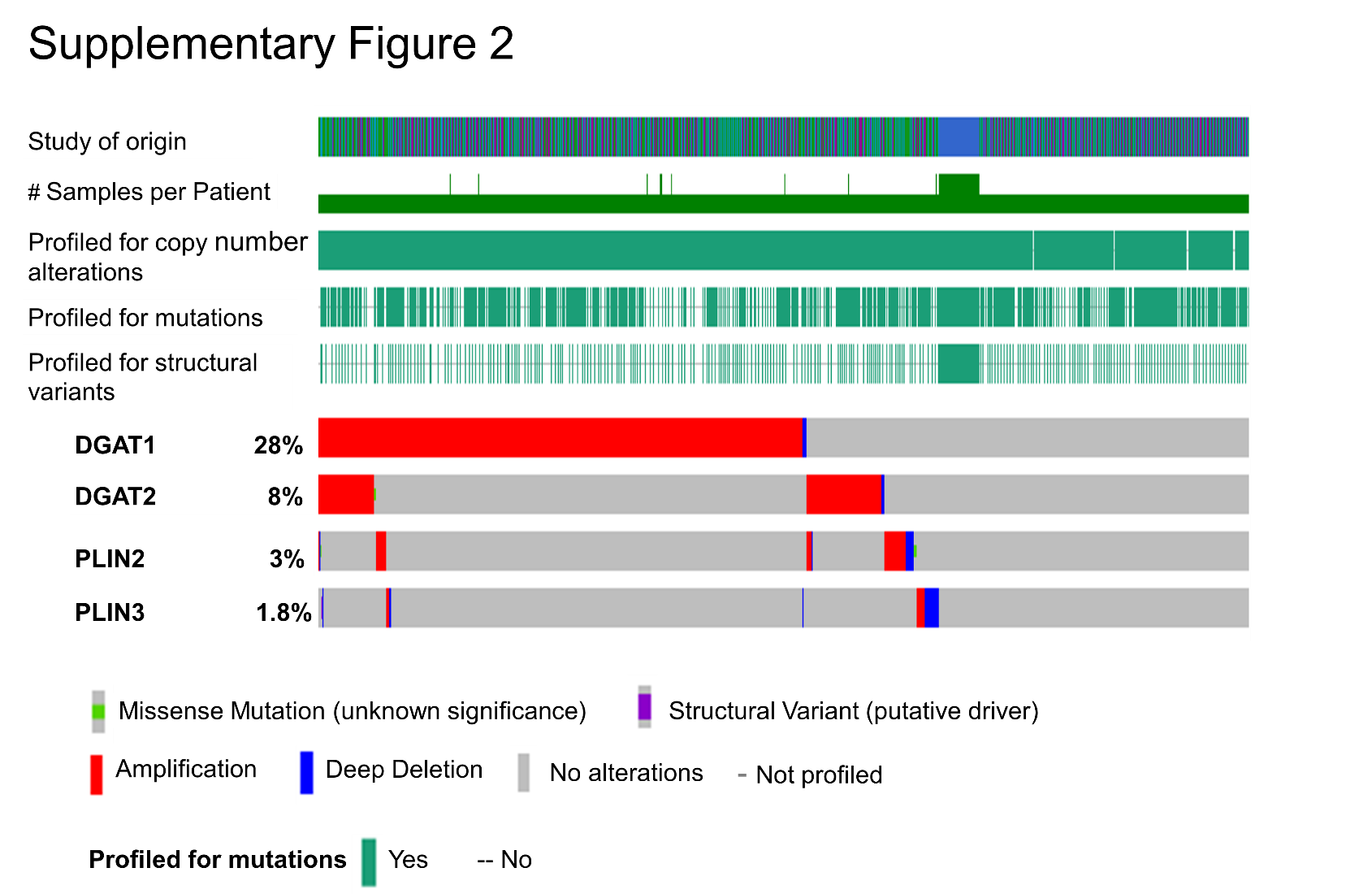


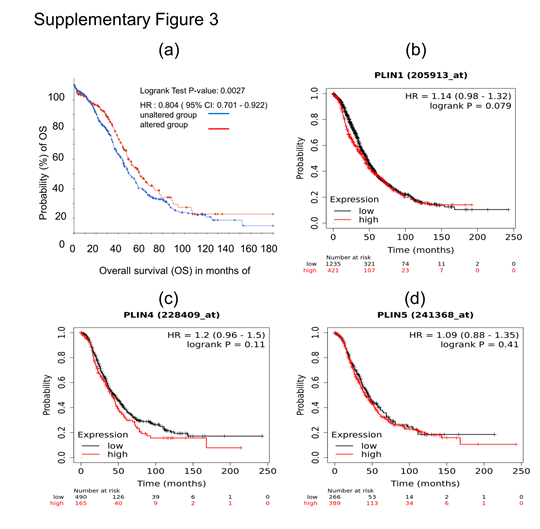

Supplement: Supplementary file 1 — Supporting Information Additional supporting information can be found online in the Supporting Information section. Table S1: Primers for RT‐qPCR targeting DGATs and PLINs. Figure S1: GEPIA2 analysis of key lipid droplet‐associated genes. The mRNA expression levels of DGAT1 (a), DGAT2 (b), PLIN1 (c), PLIN2 (d), PLIN3 (e), PLIN4 (f), and PLIN5 (g) in ovarian cancer were analyzed using the GEPIA2 platform. The expression levels of these LD‐associated genes were compared between ovarian cancer tissue (T) and normal ovarian tissue (N). The differentially expressed genes are presented in bar plots, highlighting differences in mRNA expression levels between ovarian cancer and normal ovarian tissue. ∗Significant at p < 0.05. Figure S2: DGATs oncoprint. cBioPortal “oncoprint” representation of alterations in DGATs and PLIN genes identified in ovarian cancer consisting of 1937 samples (amplification, red; deep deletion, blue; no alterations, grey). Numbers represent the combined frequency of all alterations. TCGA datasets selected are shown above. Figure S3: Survival curves of LD genes. Prognostic value of the PLIN1 (a), PLIN4 (b), and PLIN5 (c) and genes in ovarian cancer patients using the Kaplan–Meier plotter online database. (d) Prognostic value of the DGAT1 gene, comparing DGAT1 altered versus unaltered groups in ovarian cancer, using cBioportal database. [file BMRI-2025-9153643-s001.docx]
